# The Contrasting Effects of Elevated CO_2_ on TYLCV Infection of Tomato Genotypes with and without the Resistance Gene, *Mi-1.2*

**DOI:** 10.3389/fpls.2016.01680

**Published:** 2016-11-09

**Authors:** Huijuan Guo, Lichao Huang, Yucheng Sun, Honggang Guo, Feng Ge

**Affiliations:** ^1^State Key Laboratory of Integrated Management of Pest Insects and Rodents, Institute of Zoology, Chinese Academy of SciencesBeijing, China; ^2^Tourism and Air Service College, Guizhou Minzu UniversityGuizhou, China; ^3^University of Chinese Academy of SciencesBeijing, China

**Keywords:** elevated CO_2_, resistance, *Mi-1.2* gene, tomato, TYLCV, whitefly

## Abstract

Elevated atmospheric CO_2_ typically enhances photosynthesis of C3 plants and alters primary and secondary metabolites in plant tissue. By modifying the defensive signaling pathways in host plants, elevated CO_2_ could potentially affect the interactions between plants, viruses, and insects that vector viruses. *R* gene-mediated resistance in plants represents an efficient and highly specific defense against pathogens and herbivorous insects. The current study determined the effect of elevated CO_2_ on tomato plants with and without the nematode resistance gene *Mi-1.2*, which also confers resistance to some sap-sucking insects including whitefly, *Bemisia tabaci*. Furthermore, the subsequent effects of elevated CO_2_ on the performance of the vector whiteflies and the severity of *Tomato yellow leaf curl virus* (TYLCV) were also determined. The results showed that elevated CO_2_ increased the biomass, plant height, and photosynthetic rate of both the Moneymaker and the *Mi-1.2* genotype. Elevated CO_2_ decreased TYLCV disease incidence and severity for Moneymaker plants but had the opposite effect on *Mi-1.2* plants whether the plants were agroinoculated or inoculated via *B. tabaci* feeding. Elevated CO_2_ increased the salicylic acid (SA)-dependent signaling pathway on Moneymaker plants but decreased the SA-signaling pathway on *Mi-1.2* plants when infected by TYLCV. Elevated CO_2_ did not significantly affect *B. tabaci* fitness or the ability of viruliferous *B. tabaci* to transmit virus regardless of plant genotype. The results indicate that elevated CO_2_ increases the resistance of Moneymaker plants but decreases the resistance of *Mi-1.2* plants against TYLCV, whether the plants are agroinoculated or inoculated by the vector. Our results suggest that plant genotypes containing the R gene *Mi-1.2* will be more vulnerable to TYLCV and perhaps to other plant viruses under elevated CO_2_ conditions.

## Introduction

The atmospheric CO_2_ concentration, which has risen from 280 to 400 ppm since the industrial revolution, now exceeds any level in the past 65,000 years and is predicted to reach 540–900 ppm by the end of this century (IPCC, 2013). Increases in atmospheric CO_2_ alter photosynthetic rates, carbohydrate accumulation, transpiration, and other aspects of plant physiology (Ainsworth and Long, 2005; Ainsworth et al., 2008). These effects can lead to changes in the primary and secondary metabolites in plant tissue, and may therefore affect interactions between plants and pathogens, between plants and insects, and between plants, viruses, and virus vectors (Chakraborty and Datta, 2003).

The effect of elevated CO_2_ on the incidence and severity of diseases caused by plant pathogens differs among pathogens. Free-air CO_2_ enrichment (FACE) studies have indicated that elevated CO_2_ increases plant susceptibility to certain fungal species (Kobayashi et al., 2006; Melloy et al., 2010) but reduces susceptibility to certain bacterial pathogens and some fungal species (Jwa and Walling, 2001; Zhang et al., 2015). These results were largely explained by the cross-talk between jasmonic acid (JA)- and salicylic acid (SA)-signaling pathways, which are vital for plant resistance against different types of pathogens (Eastburn et al., 2011; Zhang et al., 2015). Elevated CO_2_ increased plant resistance against *Potato virus Y* in tobacco and *Tomato yellow leaf curl virus* (TYLCV) in tomato (Matros et al., 2006; Huang et al., 2012). In the field, these plant viruses are transmitted by insect vectors, most of which are sap-sucking insects (i.e., aphids and whiteflies) whose performance could be affected by elevated CO_2_ (Sun et al., 2013; Wang et al., 2014). Some aphid species exhibit increased fecundity, abundance, and survival under elevated CO_2_ (Pritchard et al., 2007; Robinson et al., 2012). In contrast, elevated CO_2_ reduced whitefly abundance at 1000 ppm but had no effect at 700 ppm (Butler et al., 1986; Tripp et al., 1992; Wang et al., 2014). It is unclear whether the effects of elevated CO_2_ on the performance of insect vectors could in turn alter virus transmission to plants.

The interactions between insect vectors and plant viruses are often assumed to be mediated by plant defenses (Belliure et al., 2005; Colvin et al., 2006; Stout et al., 2006). A growing number of studies have reported that virus infection can decrease the resistance of host plants against insect vectors. Infection of tobacco plants by Tomato Yellow Leaf Curl China Virus (TYLCCNV) suppresses JA-dependent defenses and terpenoid synthesis, thereby favoring the performance of the whitefly vector, *Bemisia tabaci*, on virus-infected plants (Zhang et al., 2012; Luan et al., 2013). Viruliferous *B. tabaci* fed more than non-viruliferous *B. tabaci* and spent more time salivating into sieve tube elements, thereby enhancing virus infection and spread (Liu et al., 2013).

*Tomato yellow leaf curl virus*, which severely damages tomato crops in many tropical and subtropical regions worldwide (Czosnek and Laterrot, 1997; Zhang et al., 2009), is mainly transmitted by the whitefly *B. tabaci* in a persistent-circulative manner (Hogenhout et al., 2008). *B. tabaci* and TYLCV have a mutualistic relationship involving their shared host plants (McKenzie, 2002; Colvin et al., 2006; Jiu et al., 2007). Thus, the interaction between *B. tabaci* and the host plant is a key determinant of TYLCV transmission and infection.

In tomato, a well-studied *R* gene, *Mi-1.2*, encodes a protein with a nucleotide-binding domain and a leucine-rich repeat region (Milligan et al., 1998). Tomato plants with *Mi-1.2* are resistant to three species of root-knot nematodes (*Meloidogyne arenaria*, *M. incognita*, and *M. javanica*) and sap-sucking insects such as whiteflies, aphids and pysllids. This gene reduces nematode or insect reproduction and abundance (Kaloshian et al., 1995; Milligan et al., 1998 ; Vos et al., 1998; Nombela et al., 2003; Casteel et al., 2006). Given that the *Mi-1.2* gene confers a moderate level of resistance to whiteflies, we suspect that the *Mi-1.2* gene might also affect TYLCV acquisition and transmission by its vectors.

In host plants not infected with virus, SA-signaling defenses reduce the feeding efficiency of viruliferous *B. tabaci*, which may subsequently affect TYLCV transmission and infection of plants (Shi et al., 2013). TYLCV infection alone can induce SA-dependent defenses, which increases the defense against subsequent feeding by *B. tabaci* (Huang et al., 2012; Shi et al., 2013). Moreover, the SA-signaling pathway is involved in *R* gene *Mi-1.2*-mediated resistance (Li et al., 2006). The transcript levels of *PR1* in the resistant *Mi-1.2* plants accumulated faster and at higher amounts than in the susceptible *mi-1.2* plants after aphid infestation (Martinez de Ilarduya et al., 2003). Thus, the regulation of the SA-signaling pathway appears to be crucial for plant resistance against both virus and vector. In tomato and other crops, the SA-signaling pathway can be modified by the environment (Huang et al., 2012; Sun et al., 2013), suggesting that environmental change could affect phytohormone SA-induced defenses in *Mi-1.2* contained plants, which may affect the severity of TYLCV and the fitness of vector *B. tabaci*.

In the current study, we assessed the effects of elevated CO_2_ on the tritrophic interactions among tomato, *B. tabaci*, and TYLCV. Two tomato cultivars were used: whitefly resistant cultivar Motelle (*Mi-1.2*) plants and its near-isogenic susceptible cultivar Moneymaker. We tested two hypotheses: (1) the *Mi-1.2* genotype of tomato would reduce TYLCV transmission and severity due to the higher resistance ability, which may indirectly suppress *B. tabaci* fitness; and (2) elevated CO_2_ would enhance plant resistance against TYLCV and *B. tabaci* by up-regulating the SA- signaling pathway.

## Materials and Methods

### Open-Top Field Chambers and CO_2_ Levels and Plants

The experiment was carried out in eight open-top field chambers (OTCs). Four of the OTCs were continuously maintained at the current ambient level of CO_2_ (about 400 ppm), and four were continuously maintained at an elevated level of CO_2_ (about 750 ppm, the predicted level by the end of this century) (IPCC, 2013). Details of the automatic control system for CO_2_ concentrations and OTCs are provided in Chen et al. (2005). Air temperature was measured three times daily (8:00, 14:00, and 18:00) throughout the experiment and did not differ significantly between the two sets of OTCs during the experiment.

Two near-isogenic tomato (*Solanum lycopersicum*) lines, the susceptible cultivar Moneymaker and the resistant cultivar Motelle (*Mi-1.2*), were used in our experiments. Motelle carries a 650-kb segment of *S. peruvianum* DNA that harbors the *Mi-1.2* gene, which makes it genetically distinct from Moneymaker (Milligan et al., 1998). These lines were selected for study due to whitefly resistance (Nombela et al., 2000). Seeds of Moneymaker and *Mi-1.2* (Motelle) plants were obtained from the National Engineer and Research Center for Vegetable, Academy of Agricultural and Forestry Sciences, Beijing, China. One week after germination, when the cotyledons were beginning to expand, the seedlings were transplanted singly into plastic pots (25 cm × 21 cm × 22 cm) containing sterilized loamy field soil (organic carbon 75 g/kg; available N 500 mg/kg; available P 200 mg/kg; available K 300 mg/kg). The pots were placed in ventilated insect-proof cages in octagonal OTCs until they grew to the 3- to 4-leaf stage. Pot placement was re-randomized within each OTC once each week. No chemical fertilizers and insecticides were used. Water was added to each pot every 2 days. Five groups of plants were used for the experiments described in the following sections (**Supplementary Figure S1**).

### Plant Growth Traits and Photosynthesis as Affected by Plant Genotype and CO_2_ Level (Group 1)

Six undamaged 8-week-old plants of each genotype in each OTC (=24 plants per treatment and 96 plants in total) were selected for measurement of photosynthetic rate and plant growth traits. The net photosynthetic rate was determined according to Guo et al. (2012) with some modification. The net photosynthetic rate of each plant was measured with a Li-Cor 6400 gas exchange system (Li-Cor Inc., Lincoln, NE, USA). The fourth mature leaf from the base of the stem was selected for measurement. All measurements were done between 9:00 and 12:00 am. The CO_2_ concentration of the incoming air was adjusted to 400 μmol mol^-1^ CO_2_ or 750 μmol mol^-1^. Relative humidity corresponded to ambient conditions. Before gas exchange was measured, photosynthetic active radiation for the leaf in the measuring cuvette was increased in steps to 1200 μmol m^-2^ s^-1^. When the CO_2_ assimilation rate was stable for at least 2 min, measurements were recorded. After that, the plants were harvested for measurement of biomass, stem diameter, and height.

### TYLCV Incidence and Disease Index as Affected by Plant Genotype, CO_2_ Level, and Agroinoculation vs. Whitefly Virus Inoculation (Group 2)

The plant–virus interactions could be affected by both plant physiology and vector transmission ability, thus, in current study, we determined the effects of elevated CO_2_ on the disease incidence and index of TYLCV by either agroinoculation or transmitted by whitefly. For agroinoculation of TYLCV, 25 8-week-old plants of each genotype in each OTC (25 plants × 4 OTC × 2 genotypes × 2 CO_2_ levels and 400 plants in total) were selected and agroinoculated as described previously (Huang et al., 2012). The TYLCV infection of tomato plants was achieved using *Agrobacterium tumefaciens*-mediated infectious inoculation (Zhang et al., 2009; Al Abdallat et al., 2010), and the infectious 2 clone (pBINPLUS-SH2-1.4A) of TYLCV- Israel [China: Shangai2] was constructed into *A. tumefaciens* strain EHA105 as described previously (Zhang et al., 2009). The infectious clone of TYLCV was provided by Professor Xueping Zhou (State Key Laboratory for Biology of Plant Diseases and Insect Pests, Institute of Plant Protection, Chinese Academy of Agricultural Sciences, China). The culture of TYLCV clone was grown in LB culture medium with kanamycin (50 μg/ml) and rifampicin (50 μg/ml) at 28°C (250 rpm) for 24 h (OD_600_ = 1.5). The bacteria culture was centrifuged for 10 min at 2500 *g* and resuspended with 50 ml buffer (10 mM MgC1_2_, 10 mM 2-(N-morpholino) ethanesulfonic acid, 200 μM acetosyringone) after which 0.2 ml of the culture was injected three times into the phloem (about 1 mm in depth) of the tomato stem at the three to four leaf stage to achieve inoculation; a sterile syringe (1 ml) with a beveled needle (0.5 mm × 20 mm) was used for injection. Inoculated plants were grown in ventilated cages in the OTCs. The incidence of TYLCV infection (percentage of plants with disease symptoms) and the disease index were determined 6 weeks after agroinoculation. Disease index values were determined as follows (Curvers et al., 2010):

(D⁢I)=Σ⁢N⁢i×R⁢i/(N×R⁢h)×100

where Ni represents the number of plants in disease symptom ranking i, Ri represents the disease symptom rank (i = 0–4), *N* represents the total number of plants investigated, and Rh represents the highest disease symptom rank. Disease symptoms were ranked mainly according to Friedmann et al. (1998): 0 = no visible symptoms: inoculated plants show the same growth and development as non-inoculated plants; 1 = very slight yellowing of apical leaf margins; 2 = some yellowing and minor curling of leaf ends; 3 = widespread leaf yellowing, curling, and cupping, with some reduction in size, but plants continue to develop; 4 = severe plant stunting and yellowing, and pronounced cupping and curling of leaves; plants stop growing.

*Bemisia tabaci* of the B biotype (Middle East Asia Minor 1, aka MEAM 1), which were kindly provided by Professor Youjun Zhang of the Institute of Vegetable and Flower, Chinese Academy of Agricultural Sciences, were reared on cabbage (non-host of TYLCV) grown in insect-proof wooden cages as previously described (Jiu et al., 2007). Viruliferous whiteflies were caged on the TYLCV-infected tomato plants in a separate greenhouse. Whiteflies from the viruliferous colony were confirmed to be infected with TYLCV prior to infestation by PCR analysis (Zhang et al., 2009). For transmission of TYLCV to tomato plants by *B. tabaci*, 60 8-week-old plants of each genotype in each OTC were randomly selected, and each of 20 plants was infested by 5, 15, or 25 viruliferous *B. tabaci* for 48 h (20 plants × 4 OTC × 2 genotypes × 2 CO_2_ levels × 3 whiteflies densities and 960 plants in total). The virus incidence and disease index of the tomato plants were determined 6 weeks after *B. tabaci* infestation.

### The Abundance and Fecundity of *B. tabaci* as Affected by Plant Genotype, CO_2_ Level, and TYLCV Infection (Group 3)

To determine the effect of *TYLCV* infection on *B. tabaci* numbers and fecundity on tomato, 16 5-week-old plants of each genotype in each OTC were randomly selected. Eight plants were agroinoculated with TYLCV, and the other eight were not. Three weeks later, we checked the TYLCV copy numbers of the new emerged leaf by qPCR and confirmed that they are all successfully infected by TYLCV. Then, 4 8-week plants from each tomato genotype and TYLCV treatment per OTC (4 plants × 4 OTC × 2 genotypes × 2 CO_2_ levels × 2 TYLCV treatment and 128 plants in total) were selected. Five newly emerged females and five newly emerged males were released onto each plant; each plant was kept in a separate whitefly proof, ventilated cage (120 mesh). After 28 days, the numbers of each developmental stage of *B. tabaci* were determined for each of the four replicates in each OTC.

To determine the effect of *TYLCV* infection of tomato on *B. tabaci* fecundity, 4 8-week plants from each tomato genotype and TYLCV treatment per OTC (4 plants × 4 OTC × 2 genotypes × 2 CO_2_ levels × 2 TYLCV treatment and 128 plants in total) were randomly selected, one mated females were introduced into each plant with a whitefly proof, ventilated cage. The females were then transferred daily to fresh leaves until they died, and the number of eggs deposited by each female was determined.

### Acquisition and Transmission of TYLCV by *B. tabaci* as Affected by Plant Genotype and CO_2_ Level (Group 4)

Forty-eight 4-week-old tomato plants were agroinoculated with the virus. Once the plants exhibited obvious symptoms 4 weeks later and were confirmed as TYLCV infected by detecting the TYLCV copies with RT-PCR in the systemic leaves according to Zhang et al. (2009), we started to inoculate whiteflies. To determine the effects of plant genotype and CO_2_ level on transmission of TYLCV by *B. tabaci*, 100 adult whiteflies were caged on the second true leaf (numbered from the apex down) of each TYLCV-infected tomato plants to obtain enough viruliferous whiteflies. After a 48-h acquisition access period, 20 viruliferous whiteflies were then caged on the second true leaf of each of four 5-week-old tomato plants (at the four-leaf stage) at three time points of each genotype in each OTC (4 plants × 4 OTC × 2 genotypes × 2 CO_2_ levels × 3 three time points and 192 plants in total) (Rubinstein and Czosnek, 1997). The whiteflies were removed after 8, 24, and 48 h inoculation access period. Infection was assessed 4 weeks later based on the appearance of TYLCV symptoms and on the number of copies of TYLCV in the leaf tissue, which was determined according to Zhang et al. (2009).

To determine the effects of plant genotype and CO_2_ level on the acquisition of TYLCV by *B. tabaci*, four six-leaf stage virus-infected tomato plants (9-week-old) of each genotype at each time point in each OTC were selected (4 plants × 4 OTC × 2 genotypes × 2 CO_2_ levels × 3 three time points and 192 plants in total); the plants had been agroinoculated about 4 weeks earlier. Before releasing whiteflies, we confirmed as TYLCV infected by detecting the TYLCV copies with PCR in the systemic leaves according to Zhang et al. (2009). Fifty adult *B. tabaci* were caged on the second true leaf (numbered from the apex down). After acquisition access periods of 2, 8, and 24 h, ten *B. tabaci* were removed from each cage, and the TYLCV copy number in each group of ten *B. tabaci* was determined.

### Quantification of Phytohormone Content, Defensive Enzyme Activity, and Defensive Gene Expression (Group 5)

For measurement of the contents of the phytohormones JA and SA and the activities of the defensive enzymes phenylalanine ammonia lyase (PAL) and lipoxygenase (LOX) in tomato plants as affected by TYLCV and CO_2_ level, four 5-week-old plants of each genotype in each OTC were agroinoculated with TYLCV; another four plants of each genotype in each OTC were not inoculated and served as controls. Four weeks later, 500 mg of leaves were collected from each plant. The leaf samples were immediately stored in liquid N until analyzed.

For measurement of expression of JA- and SA-dependent defense genes, 16 5-week-old plants of each genotype in each OTC were agroinoculated with TYLCV, and another 16 plants of each genotype in each OTC were not inoculated and served as controls. After 0, 2, 8, and 24 h, the leaves of four plants (±inoculation) of each genotype in each OTC were harvested. The leaf samples were immediately stored in liquid N until analyzed.

### Measurement of Phytohormone Content and Defensive Enzyme Activity

The contents of endogenous JA and SA in the plant leaves were measured as described by Sun et al. (2013). The activities of PAL and LOX were measured according to Guo et al. (2012).

### Real-Time Quantitative PCR of Defensive Gene Expression

For real-time quantitative PCR, each treatment sample had four technical replicates for each of the biological replications. The RNeasy Mini Kit (Qiagen, Valencia, CA, USA) was used to isolate total RNAs from tomato leaves (0.05 g from samples stored at -70°C), and about 2 μg quantities of the RNAs were used to generate the cDNAs with the QuantiTect Reverse Transcription Kit (Qiagen, Valencia, CA, USA). The mRNA amounts of four target genes were quantified by real-time quantitative PCR: proteinase inhibitor (*PI-1*), lipoxygenase (*LOX2*), phenylalanine ammonia lyase (*PAL5*), and pathogenesis-related protein (*PR1a*). Specific primers for each gene were designed from the tomato EST sequences using PRIMER5 software (**Supplementary Table S1**). The PCR reactions were performed in a 20-μL total reaction volume including 10 μL of 2x SYBRs Premix EX TaqTM (Qiagen) master mix, 5 mM of each gene-specific primer, and 1 μL of cDNA template. PCR reactions were carried out on an Mx 3000P detection system (Stratagene, USA) as follows: 5 min at 95°C; then 40 cycles of 10 s at 95°C and 20 s at 62°C; and finally one cycle of 30 s at 95°C, 30 s at 55°C, and 30 s at 95°C. A standard curve was derived from the serial dilutions to quantify the copy numbers of target mRNAs. The relative level of each target gene was standardized by comparing the copy numbers of target mRNAs with the copy number of β*-actin* (*Actin7*) (the housekeeping gene; Zhai et al., 2013), which remains constant under different treatment conditions. The β-actin mRNAs of the control were examined in every plate of PCR to eliminate systematic error.

### Statistical Analyses

All data were checked for normality and equality of residual error variances and were appropriately transformed (log or square-root) if needed to satisfy the assumptions of analysis of variance. A split-split plot design was used to analyze the univariate responses of the phytohormone contents, enzyme activities, and gene expression in plants (ANOVA, PASW Statistics 18.0, SPSS Inc., Chicago, IL, USA). In the following ANOVA model, CO_2_ and block (a pair of OTCs with ambient and elevated CO_2_) were the main effects, tomato genotype was the subplot effect, and TYLCV infection level was the sub-subplot effect:

$$X_{i\,j\,k\,\mathrm{lm}}=\mu +C_{i}+B\,{\left(C\right)}_{j\left(i\right)}+G_{k}+C\,G_{i\,k}+G\,B\,{\left(C\right)}_{k\,j\left(i\right)}+H_{l}+C\,H_{i\,l}+H\,B\,{\left(C\right)}_{l\,j\left(i\right)}+G\,H\,B\,{\left(C\right)}_{k\,l\,j\left(i\right)}+e_{m\,\left(i\,j\,k\,l\right)}$$

where *C* is the CO_2_ treatment (*i* = 2), B is the block (*j* = 4), *G* is the tomato genotype (*k* = 2), and H is the virus infection treatment (*l* = 2). *e*_m(ijkl)_ represents the error because of the smaller scale differences between samples and variability within blocks (ANOVA, SAS Institute). Effects were considered significant if *P* < 0.05. Because the effect of block and the interactive effects of block and other factors were not significant (*P* > 0.45), the effect of block and its interaction with other factors are not presented to simplify the presentation. Tukey’s multiple range tests were used to separate means when ANOVAs were significant. For analysis of the plant growth traits (biomass, stem diameter, plant height, and photosynthetic rate), TYLCV incidence and index, and the ability of *B. tabaci* to acquire and transmit TYLCV under two CO_2_ levels, a split-plot design was also applied, with CO_2_ and block as the main effects and tomato genotype as the subplot effect.

## Results

### Plant Growth Traits and Photosynthesis as Affected by Plant Genotype and CO_2_ Level (Group 1)

**FIGURE 1:**
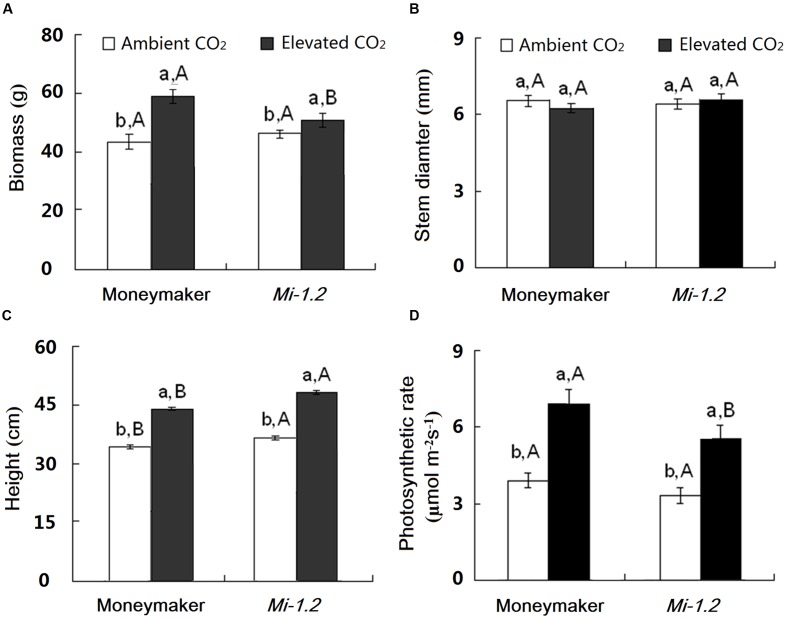
**Growth traits of two tomato genotypes (Moneymaker and *Mi-1.2*) grown under ambient CO_2_ and elevated CO_2_. (A)** Biomass, **(B)** Stem diameter, **(C)** Height, and **(D)** Photosynthetic rate. Different lowercase letters indicate significant differences between ambient CO_2_ and elevated CO_2_ within the same genotype. Different uppercase letters indicate significant differences between genotypes within the same CO_2_ treatment.

Under ambient CO_2_, growth and photosynthesis did not significantly differ between Moneymaker and *Mi-1.2* plants except for the height (**Figure 1**; **Supplementary Table S2**). Elevated CO_2_ increased biomass by 38.2%, height by 28.6%, and photosynthetic rate by 75.1% for Moneymaker plants, and increased biomass by 15.5%, height by 33.3%, and photosynthetic rate by 62.3 % for *Mi-1.2* plants. *Mi-1.2* plants had a lower biomass, a lower photosynthetic rate, and a greater height than Moneymaker plants under elevated CO_2_ (**Figures 1A,C,D**).

### TYLCV Incidence and Disease Index as Affected by Plant Genotype, CO_2_ Level, and Agroinoculation vs. Whitefly Virus Inoculation (Group 2)

**FIGURE 2:**
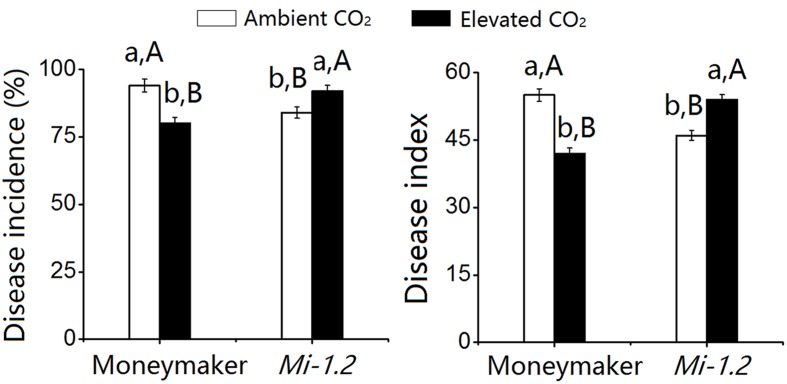
***Tomato yellow leaf curl virus* (TYLCV) disease incidence and index values in two tomato genotypes (Moneymaker and *Mi-1.2*) that were agroinoculated with the virus and then grown under ambient CO_2_ and elevated CO_2_.** Different lowercase letters indicate significant differences between ambient CO_2_ and elevated CO_2_ within the same genotype. Different uppercase letters indicate significant differences between genotypes within the same CO_2_ treatment. Means were compared with Tukey’s multiple range test at *P* < 0.05.

**FIGURE 3:**
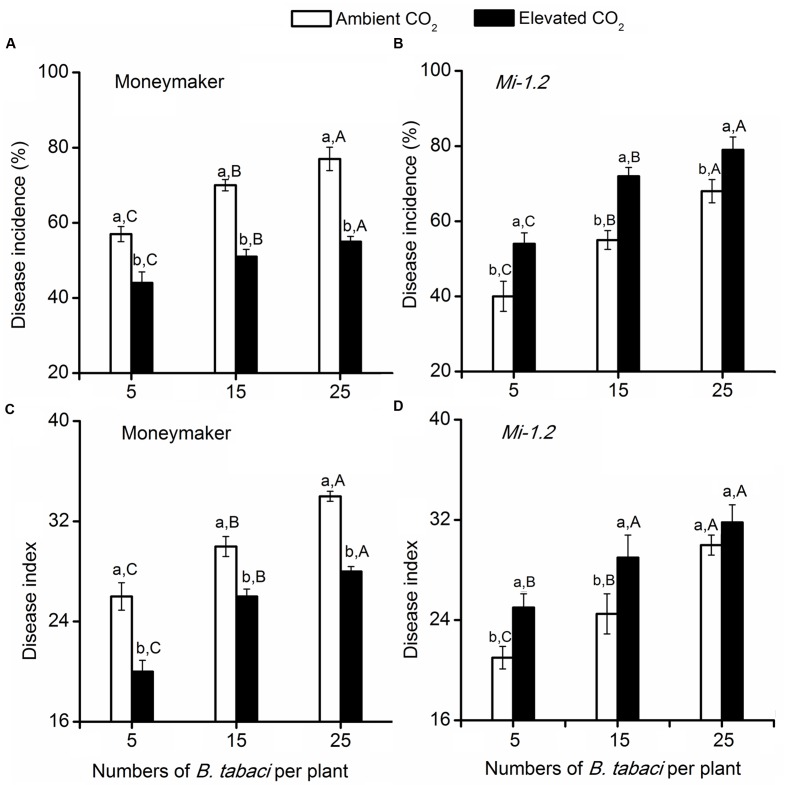
***Tomato yellow leaf curl virus* disease incidence and index values in two tomato genotypes (Moneymaker and *Mi-1.2*) that were infested with different numbers of viruliferous *Bemisia tabaci* and grown under ambient CO_2_ and elevated CO_2_. (A)** Disease incidence of Moneymaker, **(B)** Disease incidence of *Mi-1.2*, **(C)** Disease index of Moneymaker, and **(D)** Disease index of *Mi-1.2*. Different lowercase letters indicate significant differences between ambient CO_2_ and elevated CO_2_ within the same *B. tabaci* density. Different uppercase letters indicate significant differences among *B. tabaci* densities within the same CO_2_ treatment.

For the plants that were agroinoculated with TYLCV, elevated CO_2_ significantly decreased TYLCV disease incidence and index values for Moneymaker plants but increased those values for *Mi-1.2* plants (**Figure 2**; **Supplementary Table S3**). For plants that were inoculated with TYLCV by *B. tabaci*, TYLCV incidence and index values increased as the number of *B. tabaci* added increased (**Figure 3**; **Supplementary Table S4**). Elevated CO_2_ decreased the TYLCV incidence and disease index values for Moneymaker plants but increased those values for *Mi-1.2* plants when infested by the same number of viruliferous *B. tabaci* (**Figure 3**).

### Abundance and Fecundity of *B. tabaci* as Affected by Plant Genotype, CO_2_ Level, and TYLCV Infection (Group 3)

**FIGURE 4:**
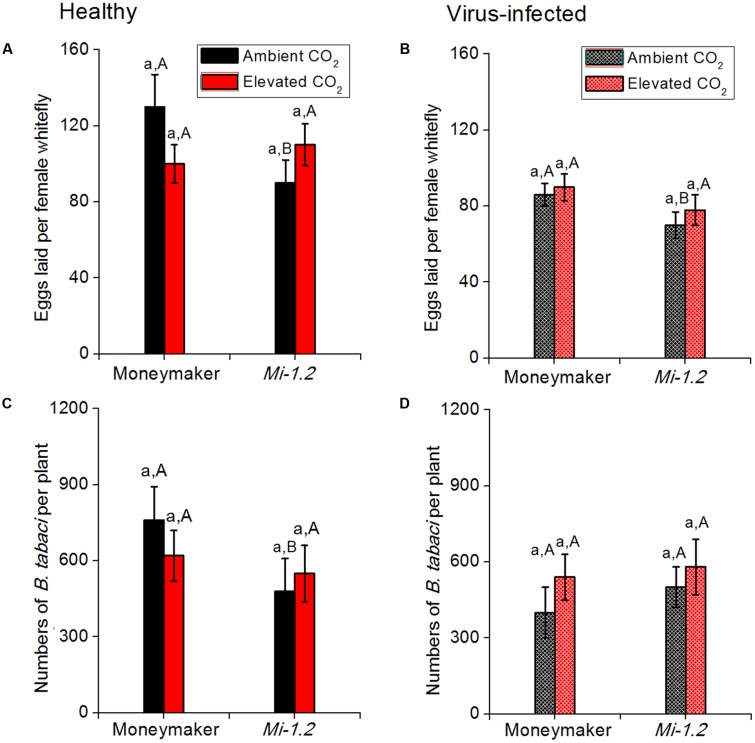
**Fecundity and abundance of *B. tabaci* on tomato plants (Moneymaker and *Mi-1.2*) that were agroinoculated or not infected with TYLCV and grown under ambient CO_2_ or elevated CO_2_. (A,B)** Fecundity of *B. tabaci* on healthy and virus-infected plants; **(C,D)** Abundance of *B. tabaci* on healthy and virus-infected plants. Different lowercase letters indicate significant differences between ambient CO_2_ and elevated CO_2_ within the same genotype. Different uppercase letters indicate significant differences in *B. tabaci* numbers within the same CO_2_ treatment.

Elevated CO_2_ did not significantly affect the abundance or fecundity of *B. tabaci* on either healthy or virus-infected plants regardless of plant genotype (**Figure 4**; **Supplementary Table S5**). Fecundity was lower on healthy *Mi-1.2* plants than on healthy Moneymaker plants under ambient CO_2_. Under elevated CO_2_, in contrast, neither *B. tabaci* fecundity nor abundance significantly differed between the two plant genotypes. *B. tabaci* abundance and fecundity were lower on TYLCV-infected plants than on healthy plants regardless of CO_2_ level or plant genotype (**Figure 4**).

### Acquisition and Transmission of TYLCV by *B. tabaci* as Affected by Plant Genotype and CO_2_ Level (Group 4)

**FIGURE 5:**
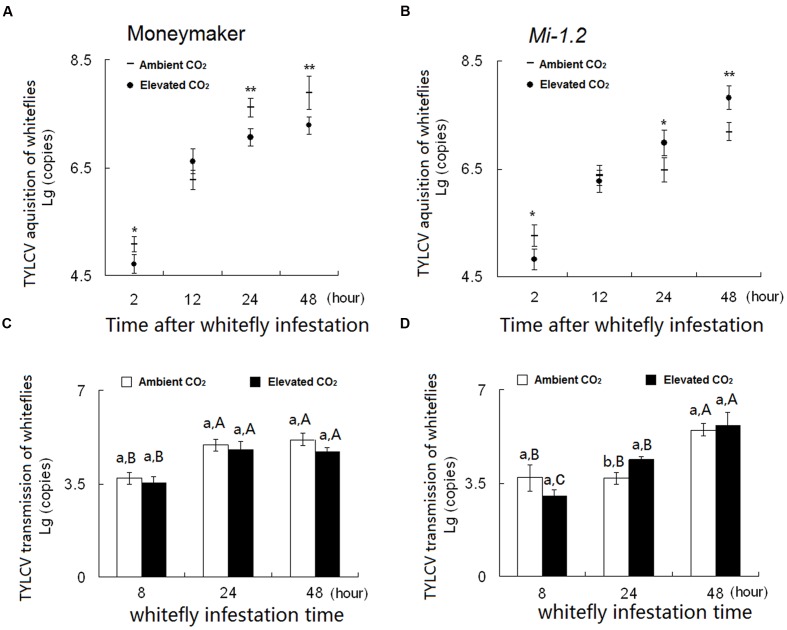
**Number of TYLCV-DNA copies acquired per *B. tabaci* when feeding on (A)** Moneymaker plants and on **(B)** *Mi-1.2* plants. Number of TYLCV-DNA copies per gram of tissue in **(C)** Moneymaker plants and **(D)** *Mi-1.2* plants infested with viruliferous *B. tabaci*. Within **(A)** and **(B)**, ^∗^ and ^∗∗^ indicate a significant difference in copy number between ambient and elevated CO_2_ at the same time point at *P* < 0.05 and 0.01, respectively. Within **(C)** and **(D)**, different lowercase letters indicate significant differences between ambient CO_2_ and elevated CO_2_ at the same time point, and different uppercase letters indicate significant differences within the same CO_2_ treatment at *P* < 0.05. In all cases, means were compared with Tukey’s multiple range test.

After whiteflies had fed on the TYLCV-infected plants for 2, 24, or 48 h, the number of TYLCV-DNA copies per *B. tabaci* was significantly lower under elevated CO_2_ than under ambient CO_2_ in the case of Moneymaker plants but the opposite was true in the case of *Mi-1.2* plants (**Figures 5A,B**). Under ambient CO_2_, *B. tabaci* contained fewer TYLCV-DNA copies when reared on TYLCV-infected *Mi-1.2* plants than on TYLCV-infected Moneymaker plants (**Figures 5A,B**). Under elevated CO_2_, *B. tabaci* contained a higher number of TYLCV-DNA copies when reared on TYLCV-infected *Mi-1.2* plants than on TYLCV-infected Moneymaker plants (**Figures 5A,B**; **Supplementary Table S6**), which is consistent with the TYLCV disease incidence and index of both genotypes before whitefly acquired TYLCV from plants (**Supplementary Figure S2**).

After viruliferous *B. tabaci* had fed on plants for 24 h, numbers of TYLCV-DNA copies in Moneymaker plants were unaffected by CO_2_ level but were higher in *Mi-1.2* plants under elevated CO_2_ than under ambient CO_2_ (**Figures 5C,D**). After a 48 h transmission access period, *Mi-1.2* plants contained fewer TYLCV-DNA copies than Moneymaker plants under ambient CO_2_ but contained higher numbers of TYLCV-DNA copies under elevated CO_2_ (**Figures 5C,D**).

### SA and JA Content and Defensive Enzyme Activity

**FIGURE 6:**
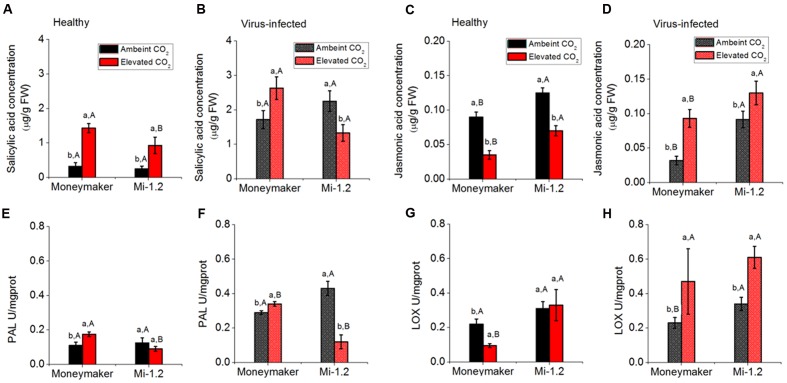
**Contents of phytohormones and activities of enzymes involved in the JA and SA signaling pathways of two tomato genotypes grown under ambient CO_2_ and elevated CO_2_ with and without TYLCV infection. (A,B)** SA concentration in healthy and virus-infected plants; **(C,D)** JA concentration in healthy and virus-infected plants; **(E,F)** PAL activity in healthy and virus-infected plants; and **(G,H)** LOX activity in healthy and virus-infected plants. Different lowercase letters indicate significant differences between ambient CO_2_ and elevated CO_2_ within the same genotype. Different uppercase letters indicate significant differences in *B. tabaci* numbers within the same CO_2_ treatment.

In Moneymaker plants that were not infected by TYLCV, elevated CO_2_ increased SA content and PAL activity but decreased JA content and LOX activity (**Figure 6**; **Supplementary Table S7**). Elevated CO_2_ increased SA content and decreased JA content of *Mi-1.2* plants (**Figures 6A,B**). After agroinoculation of TYLCV infection for 48 h, elevated CO_2_ increased the SA and JA contents and PAL and LOX activities of Moneymaker plants. In contrast, elevated CO_2_ decreased SA and PAL activity but increased JA content and LOX activity of *Mi-1.2* plants (**Figure 6**). Under ambient CO_2_, SA content and PAL activity were lower in infected Moneymaker plants than in infected *Mi-1.2* plants. Under elevated CO_2_, however, SA content and PAL activity were lower in the *Mi-1.2* plants than in Moneymaker plants regardless of TYLCV infection.

### Expression of Genes Involved in the SA- and JA-Signaling Pathways

**FIGURE 7:**
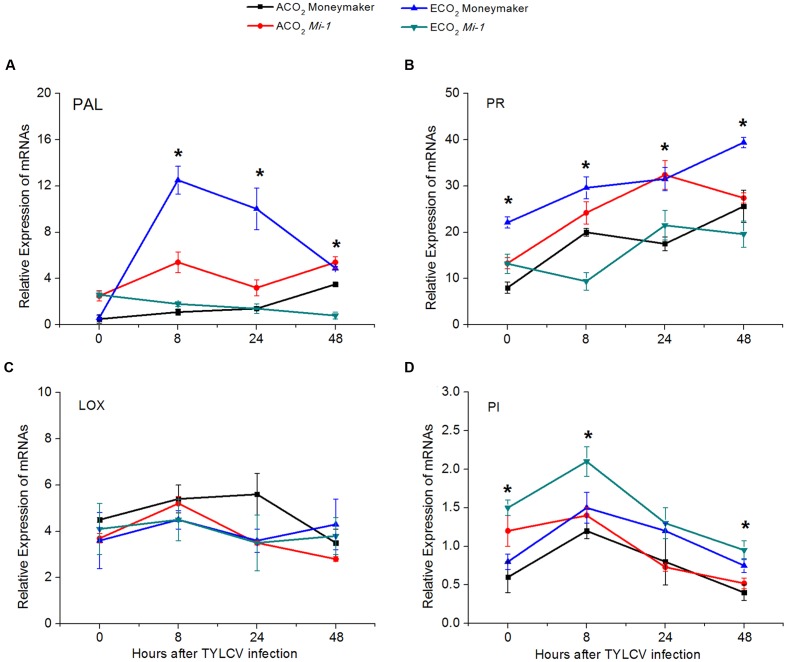
**Expression of key genes in the JA- and SA-signaling pathways of two tomato genotypes that were grown under ambient CO_2_ and elevated CO_2_ and that were infected with TYLCV for 0 to 48 h. (A)** Phenylalanine ammonia lyase (PAL); **(B)** Pathogenesis-related protein 1 (PR); **(C)** Lipoxygenase 2 (LOX); and **(D)** Proteinase inhibitor (PI). Significant differences among different treatments in the same time point at *P* < 0.05 are indicated by an asterisk.

From 8 to 48 h post-infection with TYLCV artificially, elevated CO_2_ increased the expression of genes encoding *PAL5* and *PR1a* involved in the SA-signaling pathway of Moneymaker plants but decreased their expression in *Mi-1.2* plants (**Figures 7A,B**; **Supplementary Table S8**). The expression of genes encoding *LOX2* and *PI1-1* in the JA-signaling pathway, however, was not greatly affected by elevated CO_2_ (**Figures 7C,D**; **Supplementary Table S8**). TYLCV infection tended to up-regulate the expression of genes encoding *PAL5* and *PR1a* but to down-regulate the expression of *LOX2* and *PI1-1* regardless of plant genotype (**Figure 7**). Compared with Moneymaker plants, *Mi-1.2* plants had a higher expression of genes encoding *PAL5* and *PR1a* under ambient CO_2_ but the reverse was true under elevated CO_2_ (**Figure 7**). The expression pattern of genes involved in the SA- signaling pathway across the treatments suggested that the SA-signaling pathway is an important part of plant response to TYLCV infection.

## Discussion

The *Mi-1.2* gene in tomato mediates resistance to insect vectors by triggering an array of defense responses that could in turn affect virus infection (Tameling et al., 2002). Resistance against nematodes conferred by the *Mi-1.2* gene can be reduced by elevated temperature and other environmental variables (Holtzman, 1965). In the current study, we determined the effects of elevated CO_2_ on *Mi-1.2* gene-mediated resistance against TYLCV and its vector, *B. tabaci*. Inconsistent with our hypotheses that elevated CO_2_ would increase the resistance of plants to TYLCV in both genotype, we discovered that the effects of elevated CO_2_ on TYLCV infection differed between Moneymaker and *Mi-1.2* plants. Under elevated CO_2_, the responses of the SA-signaling pathway differed between the plant genotypes, which suggested that the SA-signaling pathway may help explain the differences in plant responses to TYLCV under elevated CO_2_.

Elevated CO_2_ is expected to affect plant–virus interactions by altering both plant physiology and vector transmission ability (Malmström and Field, 1997; Rúa et al., 2013). In the present study, we found that elevated CO_2_ decreased the severity of disease caused by TYLCV on agroinoculated, Moneymaker plants, which is consistent with previous studies (Matros et al., 2006; Huang et al., 2012). The *Mi-1.2* plants, which were previously reported to be resistant to *B. tabaci* (Nombela et al., 2003), were also resistant to TYLCV, i.e., they were less diseased than the Moneymaker plants under ambient CO_2_. Under elevated CO_2_, however, the *Mi-1.2* plants had a higher disease index and severity values than wild-type plants whether they were agroinoculated with the virus or inoculated by *B. tabaci*. This result indicated that elevated CO_2_ tends to increase the resistance of Moneymaker plants but decrease the resistance of *Mi-1.2* plants against TYLCV.

In plant–virus interactions, the SA-signaling pathway is thought to provide efficient resistance against plant viruses. For example, exogenous application of SA reduces the levels of *Tobacco mosaic virus* and *Potato virus X* coat proteins in infected *Nicotiana benthamiana* leaves (Lee et al., 2011). In *N. tabacum* and *Arabidopsis*, the activation of the SA-signaling pathway inhibits the systemic movement of Cucumber Mosaic Virus (Alazem and Lin, 2015). Our results showed that tomato plants rapidly up-regulated the activity of enzymes and the expression of genes involved in the SA-signaling pathway to defend against TYLCV infection regardless of plant genotype under ambient CO_2_. The SA-signaling pathway was also found to be involved in *Mi*-mediated resistance in plants when against nematodes and aphids (Branch et al., 2004; Li et al., 2006). In the current study, *Mi-1.2* plants had a higher SA content and greater SA signaling-related enzyme activity and gene expression than Moneymaker plants under ambient CO_2_ when infected by TYLCV, which suggests that *Mi-1.2* plants have greater resistance against TYLCV infection than Moneymaker plants. Interestingly, we found that elevated CO_2_ increased SA-signaling-related enzyme activity and gene expression in virus-infected Moneymaker plants but had the opposite effect in virus-infected *Mi-1.2* plants. To our knowledge, this is the first report that the effects of elevated CO_2_ on the SA-signaling pathway differ greatly between plant genotypes differing in *R* gene-mediated resistance when those genotypes are infected by a plant virus.

Under natural conditions, TYLCV is mainly transmitted by whiteflies in a persistent-circulative, non-propagative manner (Hogenhout et al., 2008). Previous research has demonstrated that vector-borne viruses can modify vector behavior and fitness and thereby enhance virus spread by altering the host plant traits. For example, the virus could increase the nutritional quality of infected host plants, decrease the resistance of infected host plants, or increase the attractiveness of infected plants to their vectors (Jiménez-Martínez et al., 2004; Luan et al., 2013; Trêbicki et al., 2016). Infection by TYLCCNV, for example, suppresses JA-induced defenses in tomato plants, which increases the feeding and the fitness of the whitefly vector, which in turn enhances the transmission of the virus (Zhang et al., 2012). In current study, we did not observe a positive effect of TYLCV infection on *B. tabaci* performance, even though TYLCV infection suppressed JA content and the expression level of *PI* in both tomato genotypes.

Most of the insects that vector plant viruses, like aphids, whiteflies, and planthoppers, have piercing-sucking mouthparts. The piercing-sucking insects could directly suppress plant efficient defense and subsequently increase the virus transmission (Zarate et al., 2007; Walling, 2008). The fitness of sap-sucking insects could be easily affected by abiotic environment. As reviewed by Sun et al. (2016), elevated CO_2_ tends to increase the feeding efficiency of some aphids by decreasing JA-mediated resistance and by increasing nutrition content of host plants. As an exception, elevated CO_2_ decreased the feeding efficiency of *Myzus persicae* on bell pepper. Thus, the decreased performance of *M. persicae* led to a twofold decrease in virus transmission under elevated CO_2_ (Dáder et al., 2016). The current study showed that, regardless of plant genotype, elevated CO_2_ had little effect on the abundance and fecundity of *B. tabaci*. As a result, elevated CO_2_ did not affect TYLCV transmission by viruliferous *B. tabaci* regardless of plant genotype. The levels of TYLCV acquired by *B. tabaci* were positively correlated with the levels of virus in the plants (Lapidot et al., 2001). Thus, during the virus acquisition process, elevated CO_2_ decreased the numbers of TYLCV-DNA copies in *B. tabaci* feeding on Moneymaker plants but increased the numbers in *B. tabaci* feeding on *Mi-1.2* plants (**Figure 5**).

Plants have evolved sophisticated mechanisms to perceive biotic stress caused by herbivorous insects and virus pathogens (Dangl and Jones, 2001). Although tomato plants with *Mi-1.2* are resistant to sap-sucking vector whiteflies, aphids and pysllids and root-knot nematodes, the mechanisms are distinct. For instance, once infested by *B. tabaci*, the increased resistance of *Mi-1.2* prolonged the pathway stage prior to establishment of feeding site (Jiang et al., 2001). With respect to aphids, they feed for shorter periods on *Mi-1.2* plants, apparently perishing due to dehydration or starvation (Kaloshian et al., 2000). In contrast, psyllids exhibited a host selection preference and higher survival for the susceptible variety Moneymaker relative to the resistant *Mi-1.2* plants (Casteel et al., 2006). These may suggest that the effect of *Mi*-conferred resistance on different feeding stage of vector insects could further affect their virus transmission ability. In current study, although the TYLCV severity in *Mi-1.2* genotype was lower than Moneymaker, the mechanisms of defense may differ between the virus and its vector. For whiteflies, the *Mi-1.2* gene of tomato can directly recognize the elicitor and up-regulate Sgt1 (suppressor of G-two allele of Skp1) and Hsp90 (heat shock protein 90) to induce hypersensitive response (HR)-mediated effector-triggered immunity (ETI) if the same signaling mechanisms are used by *Mi-1.2* in response to aphids and whiteflies (Bhattarai et al., 2007). In contrast, the defense of *Mi-1.2* plants against TYLCV involves the up-regulation of SA-mediated resistance.

With respect to insect vectors, elevated CO_2_ may accelerate the breakdown of *R* gene-mediated resistance in *Rubus idaeus* when that plant is attacked by the aphid *Amphorophora idaei* (Martin and Johnson, 2011). In contrast, we did not find any significant effect of elevated CO_2_ on the resistance of *Mi-1.2* plants against *B. tabaci* whether the insect was feeding on virus-infected or healthy plants. With respect to the plant virus, elevated CO_2_ decreased the SA-signaling pathway of *Mi-1.2* plants and therefore decreased the resistance against TYLCV. The different response of *B. tabaci* and TYLCV to elevated CO_2_ on *Mi-1.2* plants suggests that the resistance mechanism in plants that contain *R* genes differs for pathogens vs. herbivorous insects and that those mechanisms may be respond differently to changes in the environment.

**FIGURE 8:**
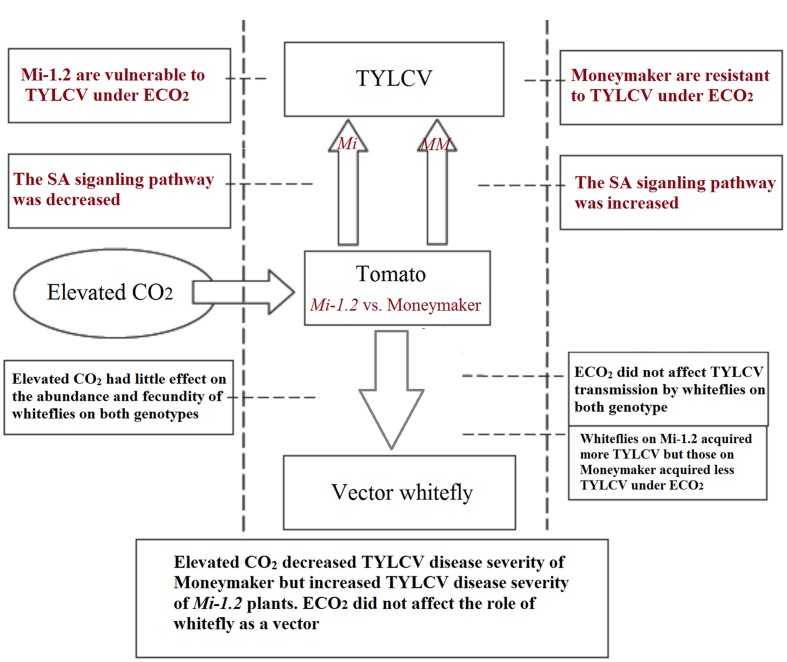
**An intergrative model to summarize major results and conclusion of this study**.

In summary, this study showed that the effects of elevated CO_2_ on TYLCV transmission and infection differed greatly between tomato genotypes with and without the *R* gene *Mi-1.2*, i.e., elevated CO_2_ decreased TYLCV disease severity of Moneymaker plants but increased TYLCV disease severity of *Mi-1.2* plants. The genotype-specific responses were closely related to the expression pattern of the SA-signaling pathway (**Figure 8**). Elevated CO_2_ did not affect the role of *B. tabaci* as a vector. The results indicate that *Mi-1.2* plants are more vulnerable than Moneymaker plants to TYLCV and may suffer greater virus damage if atmospheric CO_2_ levels continue to increase. The outcomes of this study have important implications for agricultural pest control and for transgenic breeding of resistant plants under future elevated CO_2_ conditions.

## Author Contributions

HG contribute to data analysis and article writing. LH design and do the experiment. YS wrote and revised this article. HG performed the technical work. FG conceived the project.

## Conflict of Interest Statement

The authors declare that the research was conducted in the absence of any commercial or financial relationships that could be construed as a potential conflict of interest.
