# Supplementary material for: The Contrasting Effects of Elevated CO2 on TYLCV Infection of Tomato Genotypes with and without the Resistance Gene, Mi-1.2
Source: Front Plant Sci. 2016 Nov 9;7:1680. doi: 10.3389/fpls.2016.01680 (PMC5101426; doi:10.3389/fpls.2016.01680)
Supplement: Supplementary file 1 [file Data_Sheet_1.docx]

**Figure S1** A flow diagram of the experiment design were used for the plant treatments, and sample collection, along with plant ages and time lines.

**
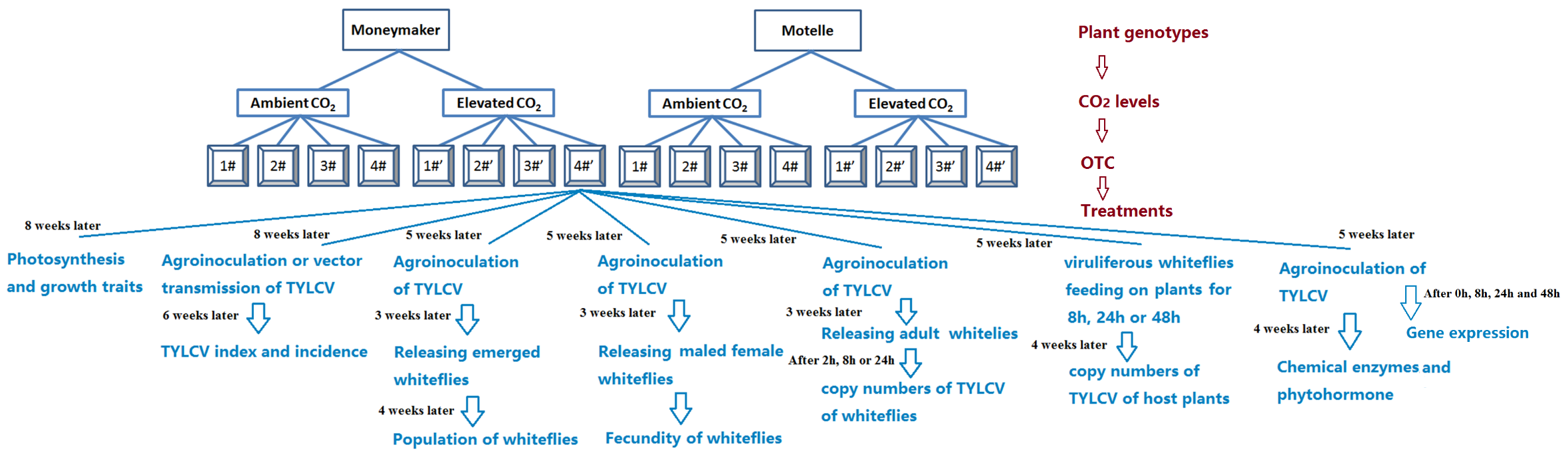
**

**Figure S2** TYLCV copy numbers in two tomato genotypes (Moneymaker and *Mi-1.2*) before releasing whitefly for TYLCV acquisition when grown under ambient CO_2_ and elevated CO_2_. Different lowercase letters indicate significant differences between ambient CO_2_ and elevated CO2 within the same genotype. Different uppercase letters indicate significant differences between genotypes within the same CO_2_ treatment. Means were compared with Tukey’s multiple range test at P < 0.05.

**
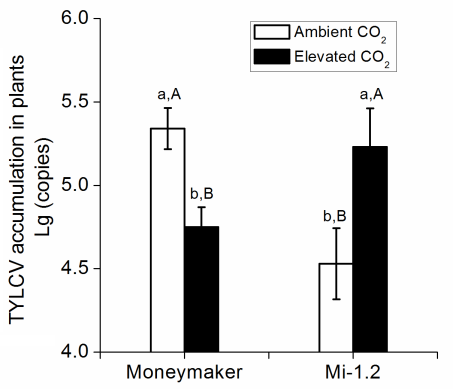
**

**Table S1.** Primer sequences used for real-time quantitative PCR.

| Gene name | Primer sequence（5’-3’） | Function |
| --- | --- | --- |
| *PI* | *F*:GCC AGA ACT TAT TGG TGT A  *R*:TGA CAT ATT GTG GCT GCT T | JA defense related |
| *LOX* | *F*:ATG GCG ACA AGA AAG ATG AGG  *R*: CTT AAA GTA GGG CGA TTA GGG | JA defense related |
| *PAL* | *F*:AGA TTG AAG TCA TTC GTG CTG  *R:*ACC CGT TGT TGT AAT AGT CGT TG | SA defense related |
| *PR* | *F*:TAC GCT ACC AAC CAA TGT G  *R*:TCC AGT TGC CTA CAG GAT C | SA defense related |
| *β-actin* | *F:* GTT GGA ATG GGT CAG AAA GAT  *R:* TTC AGT AAG CAG AAC AGG GTG | Housekeeping gene |

**Table S2**. *F* and *P* values from MANOVAs for the effect of CO_2_ level, tomato genotypes on growth traits in two tomato genotypes.

| Plant traits | Value | Treatment_(df1,df2)_^a^ | | |
| --- | --- | --- | --- | --- |
|  |  | CO_2_^b^_(1,92)_ | Geno^c^_(1,92)_ | CO_2_×Geno^d^_(1,92)_ |
| Biomass | *F* | 26.3 | 22.4 | 6.57 |
|  | *P* | <0.001*** | <0.001*** | 0.012 |
| Stem diameter | *F* | 0.901 | 1.44 | 1.21 |
|  | *P* | 0.345 | 0.234 | 0.275 |
| Height | *F* | 28.4 | 25.9 | 0.374 |
|  | *P* | <0.001*** | <0.001*** | 0.542 |
| Photosynthetic rate | *F* | 87.5 | 4.13 | 7.35 |
|  | *P* | <0.001*** | 0.045* | 0.008** |
| ^a^df1:The degrees of freedom for treatments.df2: The degrees of freedom for error.^b^Ambient CO_2_ vs. elevated CO_2_. ^c^Two genotypes of Tomato (Moneymaker vs. *Mi-1.2*).^d^ The interaction between CO_2_ level and tomato genotypes.Asterisk denotes significant P values: *<0.05, **<0.01, ***<0.001. | | | | |

**Table S3**. *F* and *P* values from MANOVAs for the effect of CO_2_ level, tomato genotype on disease severity by artificially inoculation in two tomato genotypes.

| Plant traits | Value | Treatment_(df1,df2)_^a^ | | |
| --- | --- | --- | --- | --- |
|  |  | CO_2_^b^_(1,28)_ | Geno^c^_(1,28)_ | CO_2_×Geno^d^_(1,28)_ |
| Disease incidence | *F* | 45.3 | 19.6 | 10.1 |
|  | *P* | <0.001*** | <0.001*** | 0.002** |
| Disease index | *F* | 39.4 | 10.1 | 31.7 |
|  | *P* | <0.001*** | 0.004** | <0.001*** |
| ^a^df1:The degrees of freedom for treatments. df2: The degrees of freedom for error.^b^Ambient CO_2_ vs. elevated CO_2_. ^c^Two genotypes of Tomato (Moneymaker vs. *Mi-1.2*).^d^ The interaction between CO_2_ level and tomato genotypes. Asterisk denotes significant P values: *<0.05, **<0.01, ***<0.001. | | | | |

**Table S4**. *F* and *P* values from MANOVAs for the effect of CO_2_ level, tomato genotype and whitefly density on disease incidence and index of two tomato genotypes.

| Measured indices | Value | Treatment_(df1,df2)_^a^ | | | | | | | | |
| --- | --- | --- | --- | --- | --- | --- | --- | --- | --- | --- |
|  |  | CO_2_^b^_(1,36)_ | Geno^c^_(1,36)_ | Den^d^_(2,36)_ | CO_2_×Geno^e^_(1,36)_ | CO_2_×Den^f^_(1,36)_ | | | Geno×Den^g^_(1,36)_ | CO_2_×Geno×Den^h^_(1,36)_ |
| Disease incidence | *F* | 7.19 | 4.64 | 9.63 | 18.5 | | 4.04 | 0.397 | | 4.37 |
|  | *P* | 0.011** | 0.038* | <0.001*** | <0.001*** | | 0.052 | 0.531 | | 0.041* |
| Disease index | *F* | 4.98 | 0.013 | 12.2 | 12.4 | | 5.04 | 0.031 | | 0.025 |
|  | *P* | 0.032* | 0.910 | <0.001*** | <0.001*** | | 0.031* | 0.861 | | 0.874 |
| ^a^ df1:The degrees of freedom for treatments. df2: The degrees of freedom for error.^b^Ambient CO_2_ vs. elevated CO_2_. ^c^Two genotypes of tomato (Moneymakervs.*Mi-1.2*). ^d^Whitefly density for TYLCV transmission.^e^ The interaction between CO_2_ level and tomato genotypes. ^f^ The interaction between CO_2_ level and whitefly densities.^g^ The interaction between tomato genotypes and whitefly densities. Asterisk denotes significant P values: *<0.05, **<0.01, ***<0.001. | | | | | | | | | | |

**Table S5**. *F* and *P* values from MANOVAs for the effect of CO_2_ level, tomato genotype and virus infection on whitefly population abundance and fecundity of two tomato genotypes.

| Measured indices | Value | Treatment_(df1,df2)_^a^ | | | | | | | | |
| --- | --- | --- | --- | --- | --- | --- | --- | --- | --- | --- |
|  |  | CO_2_^b^_(1,120)_ | Geno^c^_(1,120)_ | TYLCV^d^_(1,120)_ | CO_2_×Geno^e^_(1,120)_ | CO_2_×TYLCV^f^_(1,120)_ | | | Geno×TYLCV^g^_(1,120)_ | CO_2_×Geno×TYLCV^h^_(1,120)_ |
| Population^i^ | *F* | 5.09 | 9.01 | 15.6 | 3.77 | | 13.3 | 1.21 | | 5.99 |
|  | *P* | 0.028* | 0.004** | <0.001*** | 0.057 | | <0.001*** | 0.272 | | 0.015* |
| Fecundity^j^ | *F* | 4.38 | 0.978 | 21.3 | 0.480 | | 26.3 | 29.4 | | 8.44 |
|  | *P* | 0.041* | 0.327 | <0.001*** | 0.491 | | <0.001*** | <0.001*** | | 0.004** |
| ^a^ df1:The degrees of freedom for treatments. df2: The degrees of freedom for error.^b^Ambient CO_2_ vs. elevated CO_2_. ^c^Two genotypes of tomato (Moneymakervs.*Mi-1.2*). ^d^Infected or non-infected with TYLCV .^e^ The interaction between CO_2_ level and tomato genotypes. ^f^ The interaction between CO_2_ level and TYLCV infection. ^g^ The interaction between tomato genotypes and TYLCV infection. ^h^ The interaction among CO_2_ level, tomato genotypes and TYLCV infection.^i^Whitefly population abundance. ^j^ Whitefly fecundity.Asterisk denotes significant P values: *<0.05, **<0.01, ***<0.001. | | | | | | | | | | |

**Table S6**. *F* and *P* values from MANOVAs for the effect of CO_2_ level, tomato genotype and virus acquisition/transmission time on TYLCV acquisition and transmission of two tomato genotypes.

| Measured indices | Value | Treatment_(df1,df2)_^a^ | | | | | | | | |
| --- | --- | --- | --- | --- | --- | --- | --- | --- | --- | --- |
|  |  | CO_2_^b^_(1,180)_ | Geno^c^_(1,180)_ | Time^d^_(2,180)_ | CO_2_×Geno^e^_(1,180)_ | CO_2_×Time^f^_(2,180)_ | | | Geno×Time^g^_(2,180)_ | CO_2_×Geno×Time^h^_(2,180)_ |
| Acqusition^i^ | *F* | 0.853 | 0.150 | 3.85 | 9.84 | | 3.28 | 16.9 | | 7.12 |
|  | *P* | 0.357 | 0.699 | 0.023* | 0.002** | | 0.04* | <0.001*** | | 0.001** |
| Transmission^j^ | *F* | 0.144 | 8.08 | 7.93 | 7.73 | | 13.0 | 19.2 | | 15.8 |
|  | *P* | 0.705 | 0.005** | <0.001*** | 0.006** | | <0.001*** | <0.001*** | | <0.001*** |
| ^a^ df1:The degrees of freedom for treatments. df2: The degrees of freedom for error.^b^Ambient CO_2_ vs. elevated CO_2_. ^c^Two genotypes of tomato (Moneymakervs.*Mi-1.2*). ^d^Virus acquisition/transmission time . ^e^ The interaction between CO_2_ level and tomato genotypes. ^f^ The interaction between CO_2_ level and Virus acquisition/transmission time.^g^ The interaction between tomato genotypes and virus acquisition/ transmission time. ^h^ The interaction among CO_2_ level, tomato genotypes and virus acquisition/ transmission time .^i^Virus acquisition. ^j^Virus transmission. Asterisk denotes significant P values:*<0.05, **<0.01, ***<0.001. | | | | | | | | | | |

**Table S7**. *F* and *P* values from MANOVAs for the effect of CO_2_ level, tomato genotype and virus infection on phytohormone and enzymes activities in leaves of two tomato genotypes.

| Measured indices | Value | Treatment_(df1,df2)_^a^ | | | | | | | | |
| --- | --- | --- | --- | --- | --- | --- | --- | --- | --- | --- |
|  |  | CO_2_^b^_(1,24)_ | Geno^c^_(1,24)_ | TYLCV^d^_(1,24)_ | CO_2_×Geno^e^_(1,24)_ | CO_2_×TYLCV^f^_(1,24)_ | | | Geno×TYLCV^g^_(1,24)_ | CO_2_×Geno×TYLCV^h^_(1,24)_ |
| SA^i^ | *F* | 35.4 | 3.52 | 28.9 | 38.5 | | 4.88 | 0.288 | | 10.9 |
|  | *P* | <0.001*** | 0.073 | <0.001*** | <0.001*** | | 0.037* | 0.596 | | 0.003** |
| JA^j^ | *F* | 0.333 | 7.59 | 0.226 | 0.124 | | 10.9 | 0.622 | | 45.6 |
|  | *P* | 0.569 | 0.011* | 0.639 | 0.728 | | 0.003** | 0.438 | | <0.001*** |
| PAL^k^ | *F* | 14.2 | 0.023 | 12.6 | 0.011 | | 0.494 | 7.03 | | 1.14 |
|  | *P* | <0.001*** | 0.879 | <0.001*** | 0.916 | | 0.489 | 0.014* | | 0.295 |
| LOX^l^ | *F* | 16.7 | 0.592 | 0.076 | 2.38 | | 5.18 | 0.298 | | 0.859 |
|  | *P* | <0.001*** | 0.449 | 0.785 | 0.136 | | 0.032* | 0.590 | | 0.363 |
| ^a^ df1:The degrees of freedom for treatments. df2: The degrees of freedom for error.^b^ Ambient CO_2_ vs. elevated CO_2_. ^c^Two genotypes of tomato (Moneymakervs.*Mi-1.2*).^d^ Infected or non-infected with TYLCV . ^e^ The interaction between CO_2_ level and tomato genotypes. ^f^ The interaction between CO_2_ level and TYLCV infection. ^g^ The interaction between tomato genotypes and TYLCV infection. ^h^ The interaction among CO_2_ level, tomato genotypes and TYLCV infection.  ^d^ Salicylic acid. ^e^ Jasmonic acid. ^f^ Phenylalanine ammonia lyase . ^g^ Lipoxygenase.Asterisk denotes significant P values:*<0.05, **<0.01, ***<0.001. | | | | | | | | | | |

**Table S8**. *F* and *P* values from MANOVAs for the effect of CO_2_ level, tomato genotype and virus infection time on gene expression on phytohormone-mediated signaling pathwayin leaves of two tomato genotypes.

| Measured indices | Value | Treatment_(df1,df2)_^a^ | | | | | | | | |
| --- | --- | --- | --- | --- | --- | --- | --- | --- | --- | --- |
|  |  | CO_2_^b^_(1,48)_ | Geno^c^_(1,48)_ | Time^d^_(3,48)_ | CO_2_×Geno^e^_(1,48)_ | CO_2_×Time^f^_(3,48)_ | | | Geno×Time^g^_(3,48)_ | CO_2_×Geno×Time^h^_(3,48)_ |
| PAL^i^ | *F* | 10.7 | 9.78 | 6.98 | 22.9 | | 4.05 | 5.06 | | 12.2 |
|  | *P* | 0.002** | 0.003** | <0.001*** | <0.001*** | | 0.012* | 0.004** | | <0.001*** |
| PR1^j^ | *F* | 14.5 | 18.4 | 27.6 | 7.94 | | 20.9 | 3.56 | | 15.6 |
|  | *P* | <0.001*** | <0.001*** | <0.001*** | 0.007** | | <0.001*** | 0.021** | | <0.001*** |
| LOX^k^ | *F* | 0.964 | 0.93 | 6.38 | 7.94 | | 2.01 | 5.71 | | 1.55 |
|  | *P* | 0.331 | 0.34 | 0.009** | 0.005** | | 0.125 | 0.002** | | 0.213 |
| PI^l^ | *F* | 3.59 | 56.3 | 5.06 | 24.5 | | 5.71 | 26.1 | | 19.7 |
|  | *P* | 0.064 | <0.001*** | 0.004** | <0.001*** | | 0.002** | <0.001*** | | <0.001*** |
| ^a^ df1:The degrees of freedom for treatments. df2: The degrees of freedom for error.^b^ Ambient CO_2_ vs. elevated CO_2_. ^c^Two genotypes of tomato (Moneymakervs.*Mi-1.2*). ^d^Virus acquisition/transmission time . ^e^ The interaction between CO_2_ level and tomato genotypes. ^f^ The interaction between CO_2_ level and Virus acquisition/transmission time. ^g^ The interaction between tomato genotypes and virus acquisition/ transmission time. ^h^ The interaction among CO_2_ level, tomato genotypes and virus acquisition/ transmission time .^i^Phenylalanine ammonia lyase. ^j^Pathogen related protein. ^k^Lipoxygenase.^l^Proteinase inhibitor.Asterisk denotes significant P values:*<0.05, **<0.01, ***<0.001. | | | | | | | | | | |
